# Honouring the legacy of a guiding light: a tribute to Professor Antonio L’Abbate

**DOI:** 10.1093/ehjimp/qyae084

**Published:** 2024-08-14

**Authors:** Alessia Gimelli

**Affiliations:** Imaging Department, Fondazione Toscana Gabriele Monasterio, Via Moruzzi 1, 56124 Pisa, Italy

**Figure:**
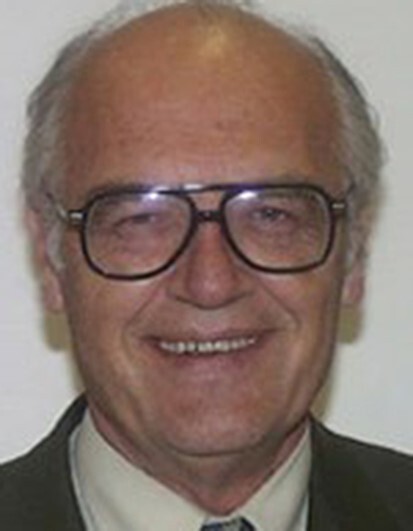


‘Remember, imagine you're speaking to people who know nothing about what you're discussing. Use simple words—simplicity is the best tool to be understood. Be direct and look into the eyes of those you're speaking to. Make sure to keep their attention, just as you would with children. Follow these tips, and your presentation will be perfect.’

I don’t know if my presentation was perfect, but I know that those words, spoken just 10 min before my thesis defence, became my mantra over the years. I’ve never forgotten them, just as I’ve never forgotten the lessons Professor L’Abbate imparted to all of us who had the privilege of working with him.

Today, we bid farewell to a luminary in the medical field, Professor Antonio L’Abbate, who passed away on 11 August 2024. As we mourn the loss of an extraordinary individual, we also take a moment to celebrate the profound impact he had on the world—not just as a pioneering researcher and esteemed physician, but, most importantly, as an exceptional human being.

Professor L’Abbate’s contributions to medicine were monumental. He was a full professor of Internal Medicine at the Sant'Anna School of Advanced Studies in Pisa, an associate researcher at the Institute of Clinical Physiology of the CNR in Pisa, the director of the Second-Level Master's Program in Underwater and Hyperbaric Medicine, and the director of the EXTREME Centre at the Sant'Anna School of Advanced Studies. His research has shaped the way we understand and treat some of the most pervasive health challenges of our time. Through countless studies, papers, and lectures, he educated a generation of doctors and inspired many more to follow in his footsteps. His work was always characterized by meticulous attention to detail, a deep understanding of complex medical issues, and an unwavering commitment to advancing scientific knowledge for the betterment of humanity.

As a physician, Professor L’Abbate embodied the ideals of the profession. He approached his patients with a blend of expertise and empathy that made him not only a skilled healer but also a comforting presence in times of distress. His dedication to his patients went beyond the walls of the clinic or hospital—he was known for his tireless efforts to ensure that everyone received the best possible care, regardless of their circumstances. In a world where healthcare can often feel impersonal, Professor L’Abbate was a beacon of compassion and kindness, reminding us all of the human side of medicine.

However, what truly set Professor L’Abbate apart was his character. He was a man of immense integrity, humility, and generosity. Despite his numerous achievements and accolades, he remained deeply grounded, always prioritizing the well-being of others above his own. He was a mentor, not just in a professional sense, but in life, offering guidance, support, and wisdom to anyone fortunate enough to know him. His warmth, humour, and genuine concern for those around him made him a beloved figure in both his professional and personal circles.

Professor L’Abbate’s legacy will endure not only in the medical breakthroughs he contributed to but also in the countless lives he touched with his humanity. He leaves behind a world made better by his presence, and while his absence will be deeply felt, his spirit will continue to inspire those who knew him, worked with him, or were simply moved by his story.

In a time when the world often seems consumed by challenges and uncertainty, the life of Professor Antonio L’Abbate serves as a powerful reminder of the good that can be achieved through dedication, kindness, and a relentless pursuit of excellence. We have lost a giant in medicine, but even more so, we have lost a truly great man.

